# Biochemical data documenting variations in mucilage polysaccharides in a range of glycosyltransferase mutants

**DOI:** 10.1038/s41597-023-02604-2

**Published:** 2023-10-14

**Authors:** Yuki Aoi, Abdelilah Benamar, Luc Saulnier, Marie-Christine Ralet, Helen M. North

**Affiliations:** 1 Université Paris-Saclay, INRAE, AgroParisTech, Institut Jean-Pierre Bourgin (IJPB), 78000 Versailles, France; 2 INRAE, UR1268 BIA, 3 impasse Yvette Cauchois, CS71627, 44316 Cedex3, Nantes, France

**Keywords:** Cell wall, Transferases

## Abstract

During Arabidopsis seed coat development, copious amounts of mucilage polysaccharides are produced in the epidermal cells. When hydrated on imbibition, these polysaccharides expand and are released to encapsulate the seed as a two-layered hydrogel. Polysaccharides are synthesized from UDP-sugars by glycosyltransferases (GTs) and several GTs, with differing activities, have been identified that contribute to mucilage polysaccharide synthesis. How these GTs orchestrate production of the complex polysaccharides found in mucilage remains to be determined. In this study, we generated a range of multiple GT mutants using either CRISPR/Cas9 targeted mutation or genetic crosses of existing T-DNA insertion mutants. Four traits for mucilage amounts or macromolecular properties were examined for four replicate seed lots from 31 different GT mutant combinations. This data provides a valuable resource for future genetic, biochemical, structural, and functional studies of the roles and properties of polysaccharides present in Arabidopsis mucilage and the relative contributions of different GTs to mucilage production.

## Background & Summary

Mucilage is formed of polysaccharides which are produced in copious amounts in seed coat epidermal cells during seed development. The polysaccharides are hydrated and expand upon seed imbibition, leading to their release from the epidermal cells and the encapsulation of the seeds as a hydrogel^1,2^. Arabidopsis mucilage is structured in two layers: an outer diffuse layer which can be removed easily by shaking with water and an inner adherent layer which remains attached to the seed coat^3^. Both layers are primarily composed of rhamnogalacturonan-I (RGI), a pectic domain. The inner layer also contains small amounts of another pectic domain, homogalacturonan (HG), together with cellulose and some hemicelluloses^1,3,4^. As Arabidopsis mucilage shares common polysaccharides with cell walls in a more accessible format, it can be used as a simple proxy to investigate polysaccharide synthesis and physico-chemical properties.

Polysaccharides are synthesized from UDP- and GDP-sugars by a range of glycosyltransferases (GTs) having different activities, some of which have been implicated in the production of mucilage pectin based on mutant phenotypes. RGI: RHAMNOSYLTRANSFERASE1 (RRT1) was the first rhamnosyltransferase demonstrated to transfer Rha from UDP-Rha onto RGI primers^5^. While *RRT1* is highly expressed in the seed coat at the developmental stage when mucilage polysaccharides are being produced, *rrt1* mutant seeds have only slightly reduced mucilage RGI amounts. Expression analysis showed that two other GTs, *MUCILAGE-RELATED70* (*MUCI70*) and *GALACTURONOSYLTRANSFERASE-LIKE5* (*GATL5*), are also highly expressed in the seed coat^6,7^. In addition to dramatically reduced mucilage polysaccharide amounts, mucilage from *muci70* mutant seeds is composed of shorter RGI polymer chains^8^. A recent study demonstrated the *in vitro* galacturonosyltransferase activity of MUCI70 towards RGI, which was subsequently named RHAMNOGALACTURONAN GALACTURONOSYLTRANSFERASE1 (RGGAT1)^9^. In contrast, seed mucilage RGI polymers in *gatl5* are larger than those of wild type (WT), even though the amount of mucilage polysaccharides is still reduced^7^. While GALACTURONOSYLTRANSFERASE11 (GAUT11) was shown to catalyse the addition of UDP-galacturonic acid (GalA) to HG primers, *gaut11* mutants surprisingly exhibited reduced amounts of RGI in mucilage.

Other GT mutants show an altered distribution of mucilage polysaccharides between the outer and the inner layers of mucilage, with no effect on global amounts. This is the case for mutants of *MUCILAGE MODIFIED 5* (*MUM5*)/*MUCI21* and *IRREGULAR XYLEM 14* (*IRX14*), which have more outer mucilage layer polysaccharides and a corresponding reduction in the inner mucilage layer^10,11^. MUM5 and IRX14 have been proposed to be xylosyltransferases that synthesize xylan, a hemicellulose, which plays a pivotal role in the formation of the inner layer by anchoring RGI to cellulose attached to the seed surface^11^. The six GT mutants cited above (*rrt1, muci70, gatl5, gaut11, irx14, mum5*) have each been shown individually to impact mucilage polysaccharide characteristics. Nevertheless, it is currently difficult to appraise how these GTs collectively contribute to RGI synthesis and the nature of the RGI-xylan relationship, due to the lack of integrated studies.

Here, we have generated double and triple mutant combinations of the six GTs indicated above by a CRISPR/Cas9 targeted gene-editing approach to create novel, knock-out mutant alleles and by genetic crosses between existing T-DNA insertion mutant alleles. Mucilage from seeds of these mutants was then analysed to create a novel data set cataloguing the amounts and macromolecular properties of the different genotypes. These data will be a rich resource for determining how different GT activities impact RGI production and modulate polymer macromolecular properties, as well as for the choice of genotypes in studies examining the role of mucilage using seeds with different amounts and properties.

**Fig. 1:**
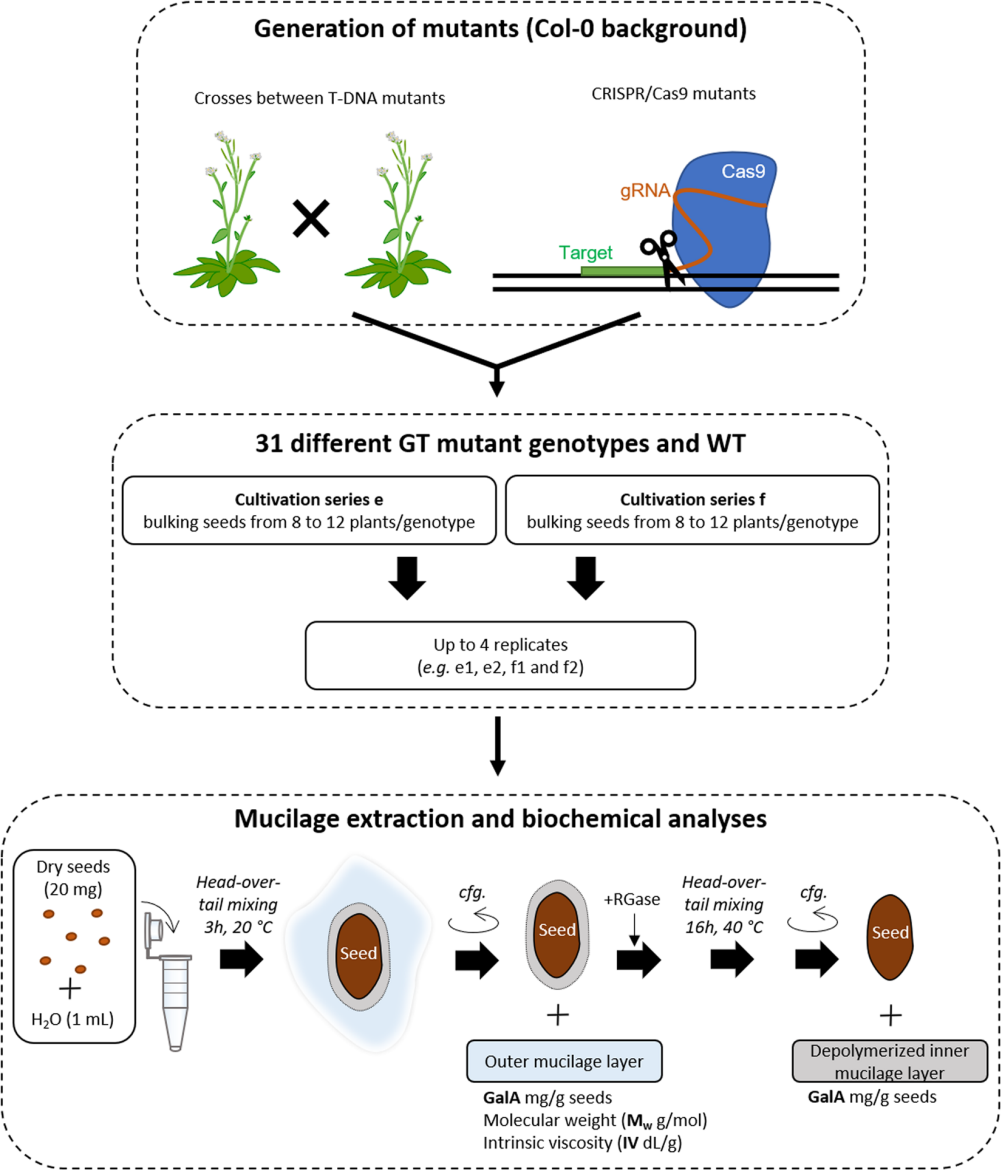
Schematic representation of the experimental workflow used to generate data records for glycosyltransferase mutant mucilage traits. Mutants were generated by either a CRISPR/cas9 based-approach or crosses between previously characterised T-DNA mutants. Seeds had been produced at two different times corresponding to cultivation series e or f, and up to four replicates were used for biochemical analyses. Analyses of mucilage GalA contents and macromolecular characteristics for the 32 genotypes are available in two data records. cfg: centrifugation; RGase: rhamnogalacturonan hydrolase.

In total, data for 31 different GT mutants and their corresponding WT (Col-0) were obtained; 11 single, 17 double and 3 triple mutants (Table 1). These were used to generate data records which correspond to mucilage amounts quantified for the outer and inner layers using a medium-throughput method, and values for macromolecular characteristics of polymers present in the outer mucilage layer. The experimental procedures and different steps in data production are summarised in Fig. 1.

**Table 1:** List of glycosyltransferase mutant genotypes used in this study and their corresponding genotype code in the dataset.

Genotype	genotype_code
WT	4000
*rrt1-1*	4001
*rrt1-2*	4002
*rrt1-3*	4003
*gaut11-4*	4004
*gatl5-1*	4005
*gatl5-3*	4006
*muci70-1*	4007
*muci70-3*	4008
*irx14-2*	4009
*irx14-4*	4010
*mum5-3*	4011
*rrt1-1 gaut11-4*	4012
*rrt1-3 gaut11-5*	4013
*rrt1-1 gatl5-1*	4014
*rrt1-2 gatl5-3*	4015
*rrt1-2 irx14-4*	4016
*rrt1-3 mum5-6*	4017
*gaut11-4 gatl5-1*	4018
*gaut11-4 muci70-1*	4019
*gaut11-4 irx14-2*	4020
*gaut11-4 mum5-3*	4021
*gatl5-1 muci70-1*	4022
*gatl5-1 irx14-2*	4023
*gatl5-3 irx14-4*	4024
*gatl5-1 mum5-3*	4025
*gatl5-3 mum5-4*	4026
*irx14-2 mum5-3*	4027
*irx14-6 mum5-4*	4028
*rrt1-2 gatl5-3 irx14-4*	4029
*gatl5-3 irx14-5 mum5-4*	4030
*rrt1-3 gaut11-5 mum5-6*	4031

## Methods

### Plant materials and growth conditions

**Fig. 2:**
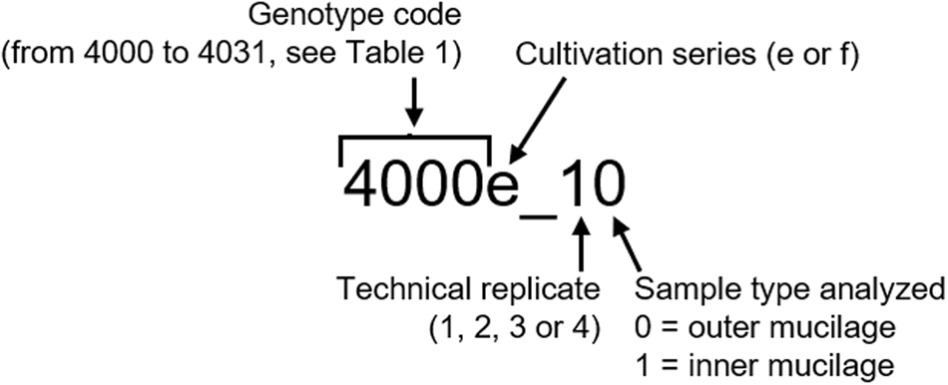
Nomenclature for seed samples analyzed to generate dataset. This is based on, and compatible with, that used in Poulain *et al*.^20^.

The *Arabidopsis thaliana* T-DNA insertion mutants *rrt1-1*, *gaut11-4, muci70-1, gatl5-1*, *irx14-2* and *mum5-3* were previously described^5–7,11^ (Table 2); all mutants are in the Col-0 accession background. The genotypes of homozygous lines, and homozygous double mutants derived from crosses between two T-DNA lines, were confirmed by PCR using the primers listed in Table 2 and Supplemental Table 1. Other mutant alleles and mutant combinations were generated by CRISPR/Cas9 targeted gene editing as described below. Surface-sterilized seeds were sown onto Arabidopsis Gamborg B5 growth medium (Duchefa; https://www.duchefa-biochemie.com/ pH 6 supplemented with 30mM Sucrose, 0.08% (w/v) bromocresol purple and 0.7% (w/v) agar. Following stratification for three days (4 °C in the dark), seeds were germinated in a growth chamber for 14 days (16 h photoperiod, 50 µmol m^−2^ s^−1^ light intensity, 60% relative humidity, 18 °C). Seedlings were transplanted to soil (Tref Substrates; https://jiffygroup.com) and transferred to the glasshouse (18 °C–28 °C, minimum photoperiod of 13 h provided by supplementary lighting) and watered for 3 weeks with tap water, then with Plan-Prod nutritive solution (Fertil; https://www.fertil.fr/). The plants were grown at two different times, series ‘e’ was grown from February 2022 to April 2022 and series ‘f’ from March 2022 to May 2022. Seeds for each genotype were harvested as bulks from eight to twelve plants from a given series (e or f) so that each series represents a biological replicate. Subsequent analyses used up to four replicates (*e.g*. e_1, e_2, f_1 and f_2, where numbers indicate the technical replicates within a series) (Fig. 2).

**Table 2:** Summary of mutant alleles studied and the primers used for their genotyping.

Mutant type	Genotype	References/ Mutation position	Primer 1	Primer 2	PCR product type
T-DNA insertion	*rrt1-1 (SALK_022924C)*	Takenaka *et al. Nature Plants* (2018)	RRT1-TF	RRT1-TR	Gene specific
			RRT1-T	SALK-LBb1	T-DNA specific
	*gaut11-4 (SAIL_406_D01)*	Voiniciuc *et al. Plant Physiol*. (2018)	GAUT11-TF	GAUT11-TR	Gene specific
			GAUT11-T	SAIL-IT-1	T-DNA specific
	*gatl5-1 (SALK_106615)*	Kong *et al. Plant Physiol*. (2013)	GATL5-TF	GATL5-TR	Gene specific
			GATL5-T	SALK-LBb1	T-DNA specific
	*muci70-1 (SALK_129524)*	Voiniciuc *et al. Plant Physiol*. (2018) Fabrissin *et al. Plant Physiol*. (2019)	MUCI70-TF	MUCI70-TR	Gene specific
			MUCI70-T	SALK-LBb1	T-DNA specific
	*irx14-2 (SALK_205399C)*	Voiniciuc *et al. Plant Physiol*. (2015) Brown *et al*. (2007)	IRX14-TF	IRX14-TR	Gene specific
			IRX14-T	SALK-LBb1	T-DNA specific
	*mum5-3/muci21-1 (SALK _041744)*	Ralet *et al. Plant Physiol*. (2016) Voiniciuc *et al. Plant Physiol*. (2015)	MUM5-TF	MUM5-TR	Gene specific
			MUM5-T	SALK-LBb1	T-DNA specific
CRISPR/Cas9 edited	*rrt1-2*	1/8 exons 37 bp deletion of G177-T213	RRT1-CF	RRT1-CR	Gene specific for sequencing
	*rrt1-3*	1/8 exons 26 bp deletion of T167-G192	RRT1-CF	RRT1-CR	Gene specific for sequencing
	*gaut11-5*	1/2 exons 1 bp insertion A between A52-G53	GAUT11-CF	GAUT11-CR	Gene specific for sequencing
	*gatl5-3*	1/1 exon 1 bp deletion of G312	GATL5-CF	GATL5-CR	Gene specific for sequencing
	*muci70-3*	1/9 exons 1 bp deletion of C79	MUCI70-CF	MUCI70-CR	Gene specific for sequencing
	*irx14-4*	1/3 exons 47 bp deletion of C385-G431	IRX14-CF	IRX14-CR	Gene specific for sequencing
	*irx14-5*	1/3 exons 1 bp insertion T between T402-G403	IRX14-CF	IRX14-CR	Gene specific for sequencing
	*irx14-6*	1/3 exons 1 bp deletion of G400	IRX14-CF	IRX14-CR	Gene specific for sequencing
	*mum5-4*	1/3 exons 1 bp insertion A between A599-T600	MUM5-CF	MUM5-CR	Gene specific for sequencing
	*mum5-6*	1/3 exons 1 bp insertion A between A194-C195	MUM5-CF	MUM5-CR	Gene specific for sequencing

For full primer details see Supplemental Table 1.

### Generation of GT mutants using a CRISPR/Cas9 targeted gene editing strategy

**Fig. 3:**
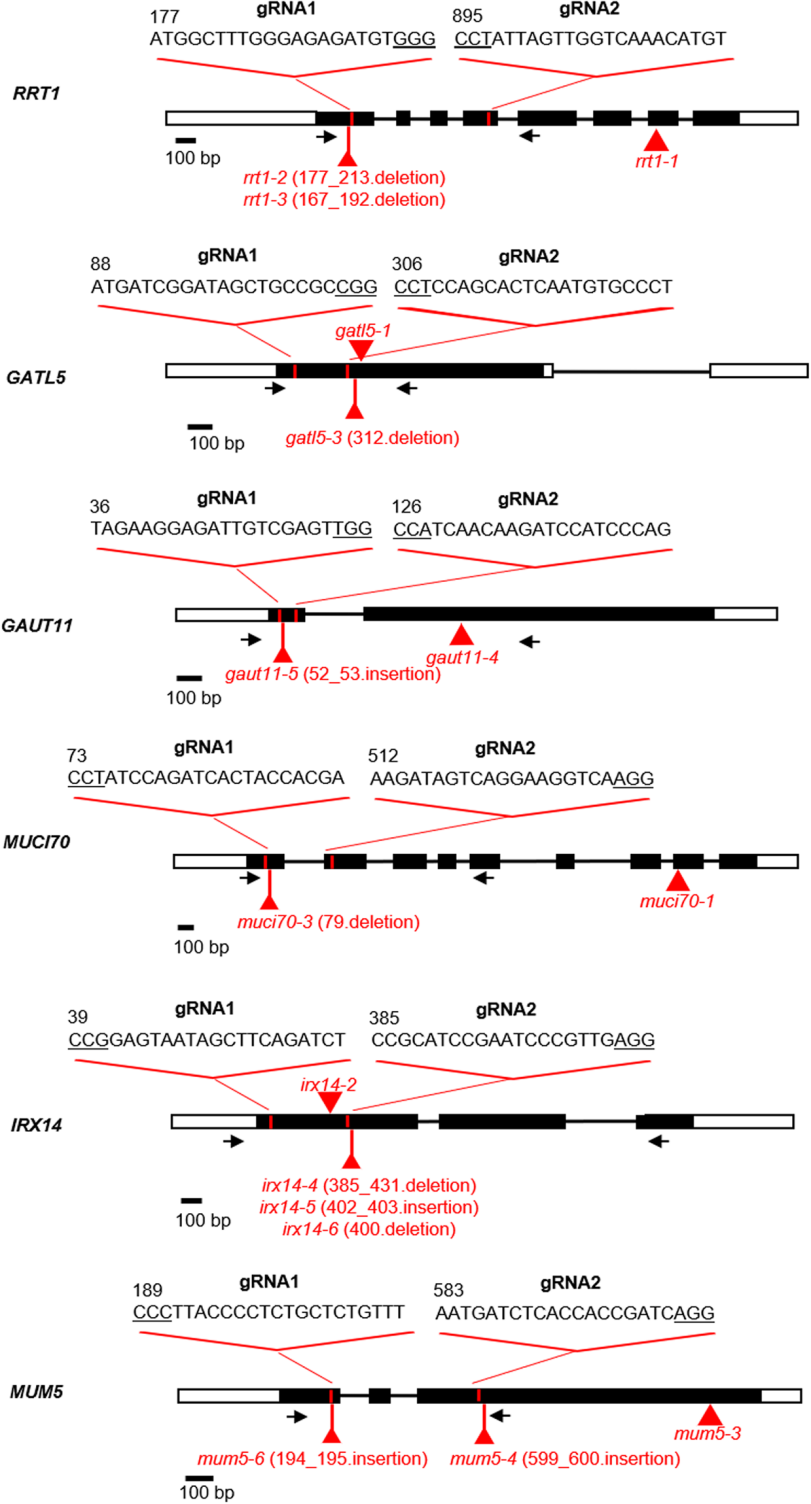
Position of mutations in the glycosyltransferase mutants used in this study and the sequence of guide RNAs used for gene-editing. Graphic representation of six glycosyltransferase genes (*RRT1*, *GATL5*, *GAUT11*, *MUCI70*, *IRX14* and *MUM5*) with black boxes representing exons and red symbols and text the site and type of mutation in the corresponding mutants. The positions of the gene-specific primers used for genotyping gene-edited mutants are indicated with black arrows. For the precise position of mutations see Table 2. Guide RNAs, gRNA; bp, base pair.

Two different guide RNAs were designed for each of the six target GTs (twelve guide RNAs in total), using the CRISPOR Web site (http://crispor.tefor.net), and selecting from proposed guide sequences based on their predicted efficiency and off-target probability. The specificity of the guides for their corresponding genes was checked by a BLAST search (https://blast.ncbi.nlm.nih.gov/Blast.cgi) against the Arabidopsis genome. Guide sequences were also selected for their position close to the 5′ end of each gene targeted, in order to increase the likelihood that induced mutations would produce truncated, inactive proteins. The target sequences of the twelve guide RNAs are listed in Table 3. Forward and reverse primers for each guide RNA were synthesized with additional sequences (Eurofins Genomics, https://eurofinsgenomics.eu/), that are complementary to the cohesive termini generated by *Bsa*I digestion of either the pUPD:*pU6-26* vector containing the Arabidopsis *U6* promoter or the pUPD:*psgRNA* vector containing a scaffold guideRNA. Both plasmids enabling the guide to be cloned directionally into the domestication vector pDGB3_α1 or pDGB3_α2, to generate a transcription unit (TU) through a GoldenBraid (GB) cyclic digestion/ligation level 0 reaction as detailed by Vazquez-Vilar *et al*.^12^. These 12 guide RNA TUs were then stacked into a single vector through a 5 level pairwise cycle that alternated omega and alpha pDGB3 vectors between levels. The transcription units pro*RPS5A:hCas9*:ter, composed of the Ribosomal Protein 5A promoter (*pRPS5A*) with the hCAS9 CDS (GB0575) and the *RbcsE9* terminator, and pro*CMV:DsRED*:ter*Nos* (gifts of Lionel Gissot), with the final 12 guide RNA TUs-pro*RPS5A:hCas9:*ter-pro*CMV:DsRED:*ter*Nos* in pDGB3_α2. The correct assembly was confirmed by PCR and sequencing at each step in *E. coli* (DH10B) or *A. tumefaciens* (C58C1) using the primers listed in Supplemental Table S1. Arabidopsis (Col-0 accession) was transformed with the final binary vector by the agrobacterium-mediated floral dip method^13^. Transgenic seeds were identified by DsRED fluorescence and DNA extracts from resulting plants subsequently genotyped for edited genes by sequencing PCR products amplified with the GT-CF and GT-CR primers (*e.g*. RRT1-CF and RRT1-CR) listed in Table 2 and Supplemental Table S1. To fix the mutation, Cas9-containing constructs were removed from segregating lines by selecting for seeds that did not exhibit DsRED fluorescence in the progeny of selected edited lines^14^. A further round of genome sequence analysis was carried out to confirm the edited mutations indicated in Fig. 3 and Table 2.

**Table 3:** Glycosyltransferase gene guideRNA target sequences.

guideRNA	Target sequence with PAM site
GATL5_guideRNA1	ATGATCGGATAGCTGCCGCCGG
GATL5_guideRNA2	CCTCCAGCACTCAATGTGCCCT
GAUT11_guideRNA1	TAGAAGGAGATTGTCGAGTTGG
GAUT11_guideRNA2	CCATCAACAAGATCCATCCCAG
IRX14_guideRNA1	CCGGAGTAATAGCTTCAGATCT
IRX14_guideRNA2	CCGCATCCGAATCCCGTTGAGG
MUCI70_guideRNA1	CCTATCCAGATCACTACCACGA
MUCI70_guideRNA2	AAGATAGTCAGGAAGGTCAAGG
MUM5_guideRNA1	CCCTTACCCCTCTGCTCTGTTT
MUM5_guideRNA2	AATGATCTCACCACCGATCAGG
RRT1_guideRNA1	ATGGCTTTGGGAGAGATGTGGG
RRT1_guideRNA2	CCTATTAGTTGGTCAAACATGT

### Mucilage Extraction and biochemical analyses

A sequential extraction of the outer and the inner mucilage layers was performed essentially as previously described^8,15^, but adapted to a much smaller quantity of seeds. Twenty mg of intact seeds were mixed head-over-tail in 1 mL of distilled water for 3 h at room temperature. The suspension was centrifuged (17,500 *g*, 10 min) and exactly 760 µl of supernatant was recovered and filtered through a disposable glass microfiber filter (13 mm diameter, 0.45 µm pore size; A.I.T. France FS13PVDF45) for analysis as an outer mucilage extract. Seeds were resuspended by adding exactly 800 µl of 50 mM sodium acetate buffer pH 4.5. Then, 10.5 μL of rhamnogalacturonan hydrolase (EC 3.2.1.171, glycoside hydrolase family 28) provided by Novozymes, at 0.1 mg/mL in 50 mM sodium acetate buffer pH 4.5, were added. The seed suspension was then incubated for 16 h at 40 °C. Samples were centrifuged (17,500 *g*, 10 min), and the supernatants were collected for analysis as inner mucilage extracts.

The GalA content of the outer and inner mucilage extracts was determined colorimetrically by the automated *m*-hydroxybiphenyl method^16^. This quantification method is based on the ability of sugars to be converted into furfuric derivatives in the presence of hot sulfuric acid. Furfuric derivatives can then condense with various phenolic compounds to produce a coloured complex that can be quantified using a spectrometer. Acidic sugars can be quantified specifically using *meta*-hydroxy biphenyl (*m*phenyl-phenol or 3 phenyl-phenol; 530 nm)^17^. GalA solutions at 20, 40, 60, 80, and 100 µg/mL were used to generate a standard curve. To take residual outer mucilage extract present in inner mucilage extract into account, GalA amounts for the inner mucilage layer were calculated by subtraction of the GalA contents corresponding to this residual outer mucilage, as detailed in the following equation:

$${\rm{GR}}\left({\rm{\mu g}}\right)=\left[{\rm{GO}}\right]\left({\rm{\mu g}}/{\rm{\mu L}}\right)\,\ast \,240\left({\rm{\mu L}}\right)$$

$${\rm{GI}}\left({\rm{\mu g}}\right)=\left[{\rm{GIE}}\right]\left({\rm{\mu g}}/{\rm{\mu L}}\right)\,\ast \,{\rm{1050}}{\rm{.5}}\left({\rm{\mu l}}\right)-{\rm{GR}}\left({\rm{\mu g}}\right)$$

where GR is the GalA contents remaining in the inner mucilage extraction, GO presents the GalA contents in outer mucilage extracts, GI presents the actual inner mucilage contents in the inner mucilage extraction, and GIE presents the GalA contents in inner mucilage extracts. The 240 µL corresponds to the volume of water added to the seed (1000 µL) after deduction of the volume recovered as outer mucilage extract (760 µL). The 1050.5 µL corresponds to 240 µL plus the volumes of buffer (800 µL) and enzyme (10.5 µL) added for adherent mucilage hydrolysis. The GalA contents for outer and inner mucilage layers were then expressed with regard to seed mass.

The outer mucilage extracts were also analyzed for their weight-average molar mass (M_w_) and intrinsic viscosity (IV) on a high-performance size exclusion chromatography (HPSEC) system (OMNISEC RESOLVE-REVEAL - Malvern Panalytical). The filtered (0.45 µm PVDF filter) outer mucilage extracts were boiled for 5 min, then samples were injected (50 µL) onto the HPSEC system maintained at 30 °C comprising a Shodex OH SB-G precolumn and a Shodex OH-Pack SB-805 HQ column eluted with 50 mM sodium nitrate at a flow rate of 0.7 mL min^−1^. Measurements were performed using a differential refractometer (OMNISEC REVEAL), a multi angle laser light scattering detector (λ = 660 nm, 44°, 60°, 76°, 90°, 108°, 124°, 140°, VISCOTEK SEC-MALS 9) and a differential pressure viscometer (OMNISEC REVEAL). Detectors were calibrated with a pullulan standard having narrow molecular mass distribution (weight-average molar mass = 40.611 Da, number-average molar mass = 38.931 Da, IV = 23.6 mL/g at 30 °C in 0.1 M sodium nitrate, refractive index increment [dn/dc] = 0.147 mL/g). Data analyses were carried out using OmniSec version 11.32 software (Malvern Panalytical) and a dn/dc value of 0.147 mL/g was used for mucilage extracts.

## Data Records

The data record contains values for GalA content (mg/g seeds) in both the outer and inner layers of mucilage, as well as the weight-average molar mass (M_w_) (g/mol) and intrinsic viscosity (IV) (dL/g) of the outer mucilage layer. The data were obtained from four replicates as described above, excepting three genotypes for M_w_ and IV and one genotype for GalA (genotypes coded 4004, 4012, 4030 and 4005, respectively, see Table 1), which had three replicates each. In addition, the four replicates analysed for the genotype coded 4010 were all from culture series f (f_1, f_2, f_3, and f_4), and for the genotype coded 4028 exclusively from culture series e (e_1, e_2, e_3, and e_4). All other genotypes were evaluated using two replicates from each culture series (e_1, e_2, f_1, and f_2) (Fig. 2). Note that certain values for M_w_ and IV were below the detection limit (B.D.L.) (genotypes coded 4007, 4008, 4018, 4019, and 4022).

**Fig. 4:**
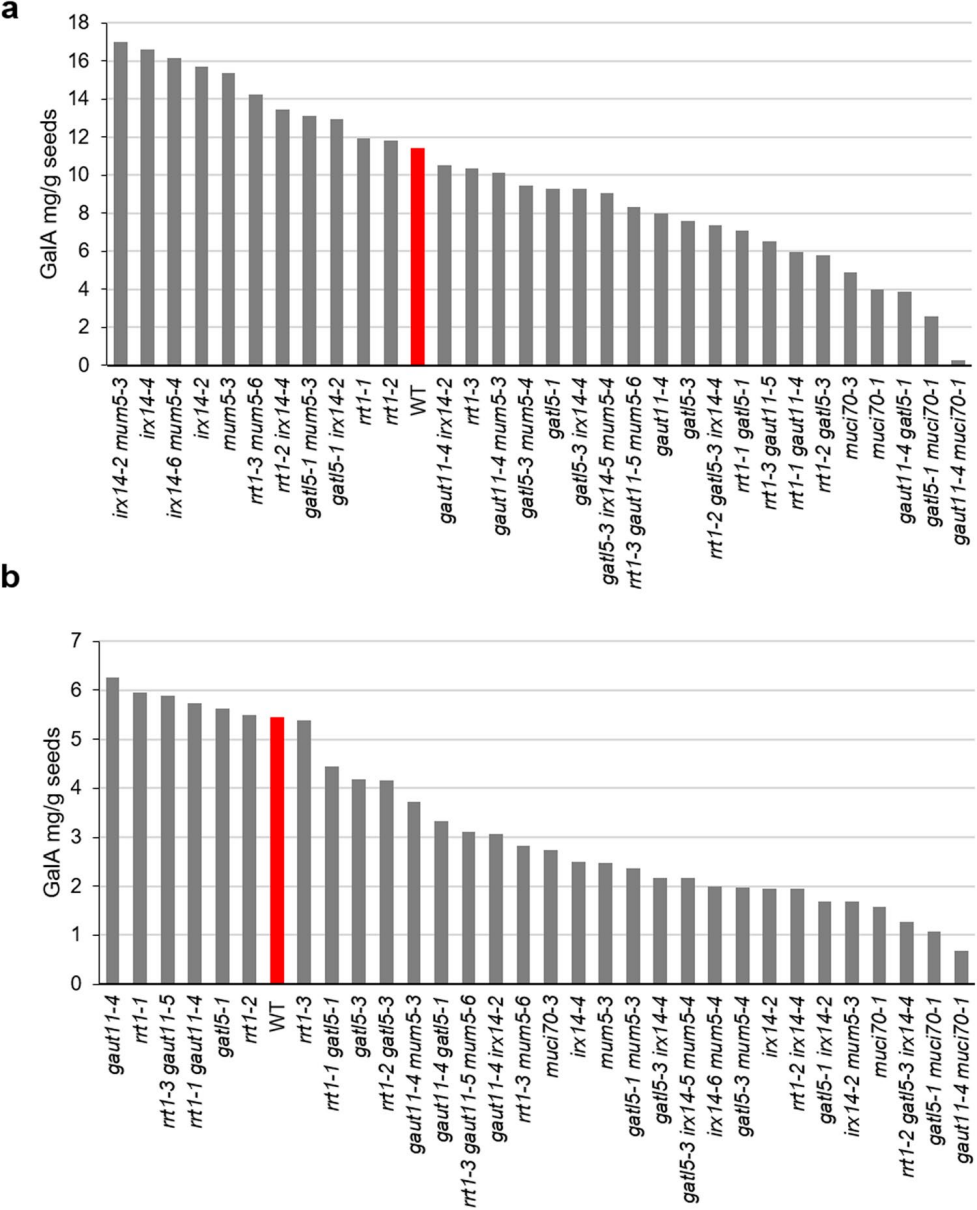
Glycosyltransferase mutants exhibit a range of GalA amounts in (**a**) outer and (**b**) inner mucilage layers. Values represent one replicate from each of the 32 different genotypes. Red bar indicates the value for wild type (WT).

The extent of variability in inner and outer mucilage GalA amounts observed between different glycosyltransferase mutants, compared to wild-type, is shown in Fig. 4 for one replicate from each of the 31 different mutant genotypes. This large diversity demonstrates the potential of these genotypes and the dataset for exploitation in future studies. The dataset is available at Data INRAE^18^. The sample nomenclature for input is outlined in Fig. 2, genotype codes in Table 1 and the description of variables in Table 4. An overview of the dataset is shown in Table 5 with the following seven columns:

1. sample_code: the sample code (see Fig. 2)
2. genotype_code: the genotype code (see Table 1)
3. cultivation_series: e or f
4. technical_replicate: the technical replicate (1, 2, 3, 4)
5. sample_type: the sample type analyzed (0 or 1) (see Fig. 2)
6. variable: the code of the variable (see Table 4 for the description)
7. value: the measured value

**Table 4:** Nomenclature used for variables in the dataset and indication of sample type analysed for each trait variable.

Variable	Unit	Variable code	Sample type analyzed
Galacturonic acid content	mg/g seeds	GalA	0 and 1
Weight average molecular weight	g/mol	M_w_	0
Intrinsic viscosity	dL/g	IV	0

Each variable measured is listed with its corresponding code, unit of measure and the sample type analyzed (for sample code see Fig. 3).

**Table 5:** Overview of the dataset comprising values for 4 seed mucilage traits from 32 genotypes.

sample_code	genotype_code	cultivation_series	technical_replicate	sample_type	variable	value
4000e_10	4000	e	1	0	GalA	11.4
4000e_20	4000	e	2	0	GalA	11.6
4000f_10	4000	f	1	0	GalA	13.4
4000f_20	4000	f	2	0	GalA	13.9
4000e_11	4000	e	1	1	GalA	5.4
4000e_21	4000	e	2	1	GalA	5.4
4000f_11	4000	f	1	1	GalA	7.1
4000f_21	4000	f	2	1	GalA	7.4
4000e_10	4000	e	1	0	M_w_	478149
4000e_20	4000	e	2	0	M_w_	445390
4000f_10	4000	f	1	0	M_w_	524358
4000f_20	4000	f	2	0	M_w_	546609
4000e_10	4000	e	1	0	IV	6.33
4000e_20	4000	e	2	0	IV	6.29
4000f_10	4000	f	1	0	IV	6.69
4000f_20	4000	f	2	0	IV	6.41

## Technical Validation

**Fig. 5:**
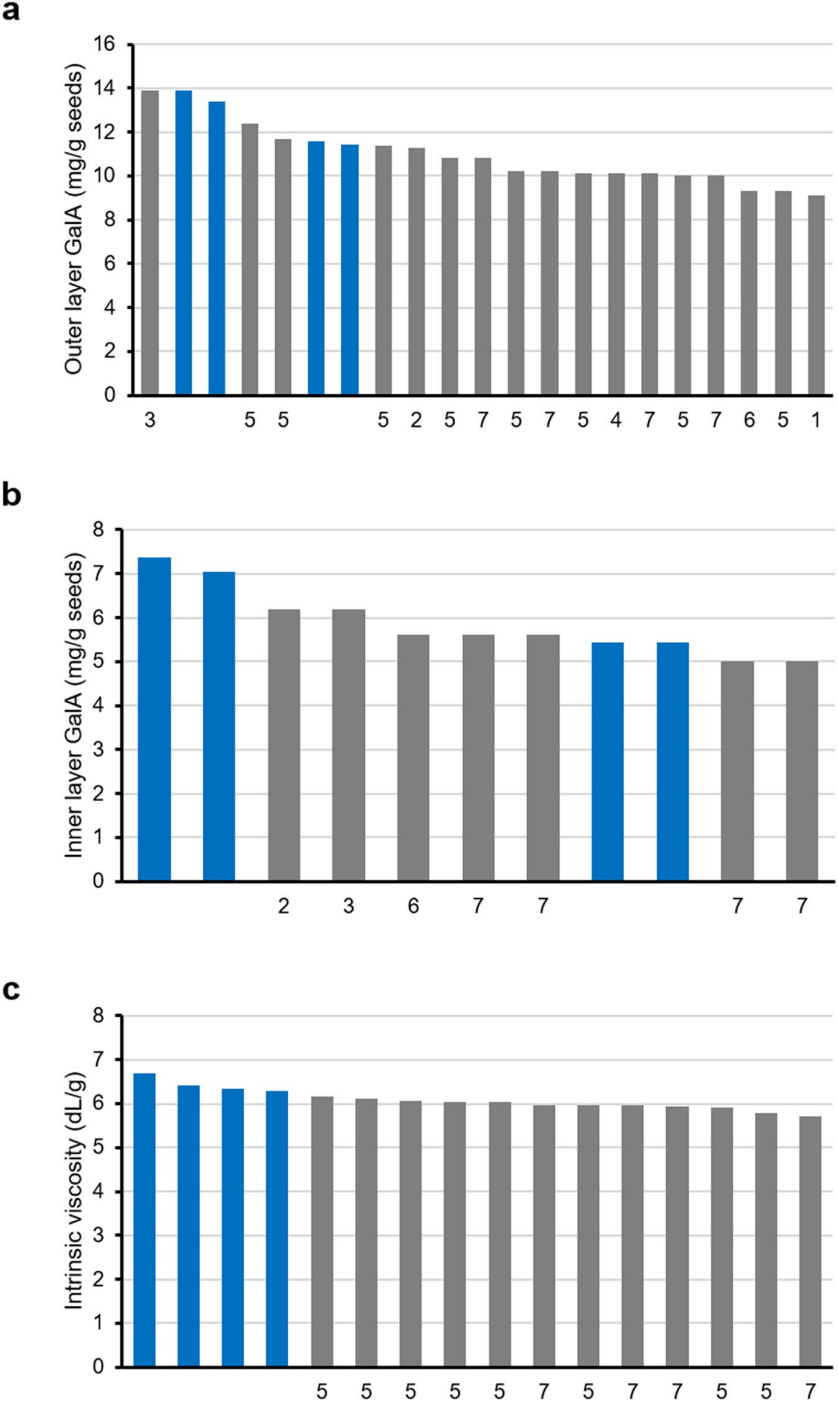
Technical validation of values obtained by comparison of values for wild-type extracts in this study to previously obtained values. GalA amounts (mg/g seed) for (**a**) outer mucilage and (**b**) inner mucilage extracts, or (**c**) intrinsic viscosity (mL/g) of outer mucilage extracts. Blue bars indicate the values for two technical replicates obtained for samples analyzed in this study (culture e or f, respectively). Grey bars indicate published values corresponding to the following references: 1, Macquet *et al*.^3^; 2, Saez *et al*.^19^; 3, Ralet *et al*.^11^; 4, Griffith *et al*.^22^; 5, Poulain *et al*.^20^; 6, Saez *et al*.^23^; 7, Cambert *et al*.^21^.

The technical quality of the dataset was validated through the use of four replicates from two different culture series, series e grown from February 2022 to April 2022 and series f grown from March 2022 to May 2022, except for four genotype/variable combinations, as indicated above. The reproducibility of results was examined for biochemical analyses based on the variation between four replicates. The variation is presented as standard errors expressed as a % of the average value of the four replicates of WT, and the highest variation observed was less than 10% (Table 6). Furthermore, values obtained for WT mucilage were similar to those previously published from independent studies, validating the reproducibility of measurements (Fig. 5)^3,11,15,19–23^. This technical validation also confirms that the calculation used to compensate for the absence of a wash step in the medium-throughput method used here produced robust inner mucilage GalA values.

**Table 6:** Variation between values from four replicates for the four traits measured in the 32 genotypes studied.

Genotype code	Outer mucilage			Inner mucilage
	GalA	M_w_	IV	GalA
4000	5.0	4.6	1.4	8.2
4001	2.3	2.1	1.1	3.2
4002	3.7	1.9	0.8	7.3
4003	4.0	3.0	0.8	3.9
4004	3.8	9.1	2.1	5.4
4005	1.4	2.7	3.1	3.4
4006	2.3	6.4	3.1	2.0
4007	1.9	—	—	4.5
4008	4.6	—	—	3.0
4009	8.0	1.3	1.2	1.2
4010	2.3	0.5	1.5	4.8
4011	5.1	1.5	1.5	4.8
4012	3.9	5.9	1.5	6.5
4013	1.5	4.8	1.6	4.2
4014	2.8	2.6	2.2	2.0
4015	2.1	5.0	3.2	2.4
4016	3.1	1.2	1.1	3.5
4017	4.0	1.3	0.3	2.4
4018	2.7	—	—	5.8
4019	0.0	—	—	4.3
4020	6.1	2.7	1.9	6.0
4021	6.8	4.9	3.3	3.9
4022	1.3	—	—	2.3
4023	3.5	2.0	1.1	4.9
4024	3.6	5.2	5.8	1.8
4025	3.4	3.2	3.1	3.1
4026	2.1	2.6	0.6	2.8
4027	7.1	1.7	1.4	2.7
4028	2.0	1.9	2.6	1.7
4029	2.7	2.0	2.4	2.3
4030	2.7	3.2	5.0	2.0
4031	3.6	1.7	1.6	4.5

Values are standard errors expressed as a % of the average value of the four replicates for wild type. GalA, galacturonic acid contents; M_w_, weight average molecular weight; IV, intrinsic viscosity. -; not calculated as below the detection limit.

For quantification of GalA concentrations, a standard curve was established both before and after a series of samples, using a serial dilution of GalA at 20, 40, 60, 80, and 100 μg/mL, to confirm technical rigour. HP-SEC columns were calibrated for IV using both a calibrant and a standard sample passed at the beginning, middle and end of a series of samples to check that no drift occurred over time.

**Table 7:** Software versions used to acquire and process data.

	Quantification of GalA amounts	Macromolecular characteristics
Signal aqcuisition	San Plus Analyzer from Skalar analytical	VISCOTEK SEC-MALS 9
		OMNISEC REVEAL - Malvern Panalytical
Signal processing	Flowaccess v3	Omnisec v11.32

### Supplementary information

Supplementary Information

## Data Availability

The different available software and the versions used to acquire and process data presented in the dataset are summarized in Table 7.
