# Supplementary material for: Biochemical data documenting variations in mucilage polysaccharides in a range of glycosyltransferase mutants
Source: Sci Data. 2023 Oct 14;10:702. doi: 10.1038/s41597-023-02604-2 (PMC10576798; doi:10.1038/s41597-023-02604-2)
Supplement: Supplementary file 1 — Supplementary Information [file 41597_2023_2604_MOESM1_ESM.pdf]

Supplemental Table S1. List of primers used in this study

Gene	Primer name	Sequence
RRT1	RRT1-G1F	5'-ATTGATGGCTTTGGGAGAGATGT-3'
	RRT1-G1R	5'-AAACACATCTCTCCCAAAGCCAT-3'
	RRT1-G2F	5'-ATTGACATGTTTGACCAACTAAT-3'
	RRT1-G2R	5'-AAACATTAGTTGGTCAAACATGT-3'
	RRT1-TF	5'-TTGTTCCGACTTTGATGGATC-3'
	RRT1-TR	5'-AGCTAACAAAACTGGCAAGGC-3'
	RRT1-T	5'-CTGGTTGTTCACATGGTTGC-3'
	RRT1-CF	5'-GGCCTCGTATGAGCTTATGG-3'
	RRT1-CR	5'-GTCGGGTATCCGTTCTGTTC-3'
GATL5	GATL5-G1F	5'-ATTGATGATCGGATAGCTGCCGC-3'
	GATL5-G1R	5'-AAACGCGGCAGCTATCCGATCAT-3'
	GATL5-G2F	5'-ATTGAGGGCACATTGAGTGCTGG-3'
	GATL5-G2R	5'-AAACCCAGCACTCAATGTGCCCT-3'
	GATL5-TF	5'-ACACTCCCCTCTCTCTCTCAC-3'
	GATL5-TR	5'-TCCATTTCTCAATTCGTTTCG-3'
	GATL5-T	5'-GCAAATCACGGCAACTACCTC-3'
	GATL5-CF	5'-CCGCATTAGCCATGATTCTC-3'
	GATL5-CR	5'-TAGGGCTTGTCTCACGGAAG-3'
GAUT11	GAUT11-G1F	5'-ATTGTAGAAGGAGATTGTCGAGT-3'
	GAUT11-G1R	5'-AAACACTCGACAATCTCCTTCTA-3'
	GAUT11-G2F	5'-ATTGCTGGGATGGATCTTGTTGA-3'
	GAUT11-G2R	5'-AAACTCAACAAGATCCATCCCAG-3'
	GAUT11-TF	5'-GTTCTGCTCGAAATCGTGGT-3'
	GAUT11-TR	5'-TTCCAAAGCGTTCGTTCTCT-3'
	GAUT11-T	5'-TTCCAAAGCGTTCGTTCTCT-3'
	GAUT11-CF	5'-GTTCTGCTCGAAATCGTGGT-3'
	GAUT11-CR	5'-TTCCAAAGCGTTCGTTCTCT-3'
MUCI70	MUCI70-G1F	5'-ATTGTCGTGGTAGTGATCTGGAT-3'
	MUCI70-G1R	5'-AAACATCCAGATCACTACCACGA-3'
	MUCI70-G2F	5'-ATTGAAGATAGTCAGGAAGGTCA-3'
	MUCI70-G2R	5'-AAACTGACCTTCCTGACTATCTT-3'
	MUCI70-TF	5'-CCCTTATTCTGTCGCAAAGC-3'
	MUCI70-TR	5'-CCGTCTGTCTCTTCCTCGTC-3'
	MUCI70-T	5'-CCGTCTGTCTCTTCCTCGTC-3'
	MUCI70-CF	5'-GACTGGATTGGGTGTTCGAT-3'
	MUCI70-CR	5'-CATCAAGGCCTCGTTCTCTC-3'
IRX14	IRX14-G1F	5'-ATTGAGATCTGAAGCTATTACTC-3'
	IRX14-G1R	5'-AAACGAGTAATAGCTTCAGATCT-3'
	IRX14-G2F	5'-ATTGCCGCATCCGAATCCCGTTG-3'
	IRX14-G2R	5'-AAACCAACGGGATTCGGATGCGG-3'
	IRX14-TF	5'-GCAGCAACAACAGCAAGAAG-3'
	IRX14-TR	5'-CCTTGGGATAATGGCATTTG-3'
	IRX14-T	5'-GCAGCAACAACAGCAAGAAG-3'
	IRX14-CF	5'-GCAGCAACAACAGCAAGAAG-3'
	IRX14-CR	5'-CCTTGGGATAATGGCATTTG-3'
MUM5	MUM5-G1F	5'-ATTGAAACAGAGCAGAGGGGTAA-3'
	MUM5-G1R	5'-AAACTTACCCCTCTGCTCTGTTT-3'
	MUM5-G2F	5'-ATTGAATGATCTCACCACCGATC-3'
	MUM5-G2R	5'-AAACGATCGGTGGTGAGATCATT-3'
	MUM5-TF	5'-GGAATTGCGCTAAGAGATCG-3'
	MUM5-TR	5'-CCTTTGTGAATTGCCATCCT-3'
	MUM5-T	5'-CCTTTGTGAATTGCCATCCT-3'
	MUM5-CF	5'-GGAATTGCGCTAAGAGATCG-3'
	MUM5-CR	5'-TTGAGCTCAGGGATGGTTTC-3'
GoldenBraid cloning	pDGB3-F	5'-TTGTGCCGAGCTGCCGGTCG-3'
	pDGB3-R	5'-CCTTTTCACGCCCTTTTAAATATCCG-3'
	LG982hCas9-R	5'-CTTTTCATGGTACGCCACCT-3'
	LG1256prRPS5A-R	5'-GGCAGTGTCAAATGCAGAAA-3'
	LG989hCas9-F	5'-GAAAAGGAACAGCGACAAGC-3'
	prCMV.S2.R	5'-TGGTTTCGATCCACTTTCTTACAAATTTCTC-3'
T-DNA	SALK-LBb1.3	5'-ATTTTGCCGATTTCGGAAC-3'
	SAIL-LB1	5'-GCCTTTTCAGAAATGGATAAATAGCCTTGCTTC-3'
